# Autonomy and compulsory care in the Netherlands

**DOI:** 10.1017/S1092852925100370

**Published:** 2025-09-19

**Authors:** Esther Nauta, Gerben Meynen

**Affiliations:** Willem Pompe Institute for Criminal Law and Criminology and Utrecht Centre for Accountability and Liability Law (UCALL), Faculty of Law, Economics and Governance, Utrecht University, Utrecht, The Netherlands

**Keywords:** Mental illness, compulsory care, The Netherlands, autonomy, community care

## Abstract

This chapter discusses the Dutch Law on Compulsory Mental Healthcare (Wvggz), which aims to strengthen the autonomy of patients with severe mental disorders by recognizing that coercive measures can be used not only to prevent harm, but also to restore autonomy. This approach challenges the traditional notion that coercive measures inherently undermine autonomy. The chapter also explores the unintended consequences of the law, such as increased bureaucracy. We argue that while the Wvggz introduces valuable ideas, its practical implementation has highlighted the challenges of translating legislative goals into effective practices.

## Introduction

In the Netherlands, compulsory care of patients with mental disorders such as schizophrenia is regulated by the Law on Compulsory Mental Healthcare^1^ (*Wet verplichte geestelijke gezondheidszorg* or Wvggz). This law replaces its predecessor, the Law on Special Admissions to Psychiatric Hospitals (*Wet bijzondere opnemingen in psychiatrische ziekenhuizen* or Bopz). The legislative process for this new law took over a decade. Discussions in parliament, with clinicians and in civil society focused much on the complexity of the relevant legal procedures and on the autonomy and legal position of the patients. In this paper, we discuss the Wvggz, its background, and the central procedures for the judicial authorization of compulsory care. We also discuss which forms of compulsory care the Wvggz allows—as the Wvggz provides options for compulsory care in mental health clinics but also at home and in community settings. While the Wvggz is a complex law with many elements, in this paper, we focus on various ways the Wvggz is purportedly aimed at *enhancing autonomy* of patients with severe mental illness. In conclusion, we show how Dutch regulations aimed at enhancing autonomy also create more complexity and bureaucracy for patients.

## Background to the law on compulsory mental healthcare

As of 2020, the Wvggz has replaced the 1994 Law on Special Admissions to Psychiatric Hospitals. The Bopz had been split into two mental regimes, one for patients with mental disorders (Wvggz) and one for patients with mental disabilities or psychogeriatric disorders (Wzd). Under the regime of the Bopz, all compulsory care for mental health patients had to take place in psychiatric hospitals. As such, the Bopz was a hospitalization regime. This was considered not to be sustainable and parliamentary efforts for more modern legislation started as early as 2008. The focus of the new regulations was that compulsory psychiatry no longer had to involve the forced hospitalization of the patient in all cases. Under the new system, other forms of compulsory care, such as administering forced medication to the individual at home, would also become possible. Rather than a hospitalization regime, the new legal framework would provide a regime for (coercive) care more broadly.

Moreover, the new legislation, through a distinction between two separate laws, was intended to ensure treatment that is better tailored to the specific patient group.

During the legislative process, much emphasis was placed on the legal position or protection of the patients. Through new kinds of procedures and more communication toward the patients and their relatives, the new legal framework was supposed to enhance patient autonomy, which we discuss further in Section “Compulsory care and autonomy” below.

During the lengthy legislative process, strong criticism was repeatedly voiced regarding the proposals for new legislation. Practitioners and lawyers alike warned that these laws would cause implementation issues (including a lot of bureaucracy) and allow for excessive intrusions into the patient’s personal privacy. Nevertheless, the new legislation entered into force in January 2020. Since then, the Wvggz has been amended multiple times a year. It has been called a “bureaucratic monster” but it is also considered to be valuable.^2^

A central goal of the introduction of the new legal regime was to reduce coercive care.^3^ However, as their analysis of the data so far shows, such a reduction in coercive measures has not yet taken place.

## The Wvvgz and the authorization for compulsory care

### Procedure

As many politicians, clinicians, and lawyers have noted, the Wvggz is very complex and difficult to comprehend even for lawyers and seasoned practitioners.^4^ There are basically two procedures. The first procedure is for very urgent situations where immediate action needs to be taken. Here, it is not a judge but the mayor who authorizes (temporary) compulsory care. In the second, regular procedure, it is the judge who gives an authorization (*zorgmachtiging*). Although compulsory care for the interest of the patient and/or interest of society is substantively, of course, a matter of public law, in the Netherlands, it is the civil judge who decides whether such authorization might be given. To make matters more unordinary, it is the ‘civil’ public prosecutor who petitions the civil judge for an authorization.

The Wvggz requires a complicated and elaborate preparation of a petition for an authorization for compulsory care of mental health patients.^5^ As soon as the public prosecutor starts with the preparation of a petition, they appoint a medical director (*geneesheer-directeur*). The medical director is a psychiatrist affiliated with a mental health care institution who is responsible for the legal procedures regarding compulsory care. The medical director then appoints an independent psychiatrist who must see the patient and draft a medical statement about the mental health of the individual involved. The psychiatrist has access to relevant information, such as police and judicial data. The medical statement must mention which symptoms the individual exhibits, the (preliminary) diagnosis of the mental disorder, the relationship between the disorder and the behavior leading to the risk of serious harm, and, specifically, what forms of compulsory care are needed to reduce the risk of harm. Several types of disorders are distinguished in the form. The form, for example, includes neurobiological developmental disorders (such as autism), schizophrenia spectrum disorders and other psychotic disorders, and bipolar mood disorders. A study of compulsory admissions in Amsterdam during 15 years under the old legal regime of the Bopz showed that 32.0% of the patients were diagnosed with schizophrenia and 30.5% with other psychotic illnesses.^6^ We expect that under the new law, in principle, the percentages of psychotic illness in general and schizophrenia in particular are likely to be similar, also because there have not been drastic changes in the number of people receiving compulsory care.^3^

In addition to a psychiatrist, the medical director also appoints a care manager (*zorgverantwoordelijke*). This care manager establishes a proposal for a care plan, which outlines the compulsory treatment the patient should receive. After the medical director has determined that the proposed care plan meets the legal requirements of the Wvggz, they pass on their findings and the proposal for a care plan to the public prosecutor. In the petition, the public prosecutor substantiates which forms of compulsory care should be included in the care authorization. Unlike the regime of the Bopz, which only provided for compulsory hospitalization, the Wvggz allows for outpatient care as well. This could include administering medication and supervising the patient in their home or in community care. The forms of compulsory care that can be included in the authorization are listed in the Wvggz. Examples are administering fluid, food, and medication. Notably, patients have the possibility to write their own plan (*plan van aanpak*) in order to avoid compulsory care. If the patient makes use of this possibility, the independent psychiatrist has to take this alternative plan into account and to evaluate its feasibility. Based on the petition and the accompanying information, eventually the judge decides on the authorization for compulsory care. The decision-making framework for the judge is discussed in the next paragraph.

### Criteria

When deciding whether to authorize compulsory care, the Wvggz stipulates that the judge tests three central criteria. Firstly, the patient must suffer from a *mental disorder.* Secondly, there must be a *risk of harm* either to the person themselves, other people or both. This risk has to be a result of the mental disorder. Thirdly, the proposed forms of compulsory care have to be *proportionate.* This means that there are no other options to reduce the risk of harm and there are no options for voluntary treatment (necessity), it is reasonably expected that the compulsory care will be effective (suitability) and giving compulsory care is proportionate considering the goal of preventing harm (proportionality). The Wvggz explicitly states that compulsory care has to be a *last resort.*

## Compulsory care and autonomy

The Wvggz regime has a few components that are meant to enhance the autonomy of the patient. Some of these were newly introduced with the Wvggz, while others existed under the previous regime of the Bopz as well. Firstly, the possibility of home and community treatments—rather than hospitalization in all cases—is meant to allow for more proportionate and less intrusive forms of compulsory care. This possibility is new and was a core aspiration of the Wvggz.

Secondly, the Wvggz includes the possibility for self-binding directives (SBD). Through an SBD, a patient can give advance consent to specific forms of mental healthcare during a mental health crisis.^1^ The Netherlands is one of the very few jurisdictions in the world with provisions for SBDs for mental health patients.^7^ SBDs are aimed at enhancing patient autonomy by giving them the opportunity to decide for themselves what kind of compulsory healthcare they consider to be beneficial in certain scenarios.

Thirdly, the wishes and opinions of a patient have to be respected (*wilsbekwaam verzet)*, unless they are incompetent, their life is in danger, or they pose a serious risk to others. In this way, patients can be (co)decision-makers about their own treatment.

Furthermore, the Wvggz stipulates what should be the *purpose* of the compulsory care, and the relevant purposes need to be specified in the proposal for the care plan. In this regard, the Wvggz explicitly mentions restoring the mental health of the person concerned in such a way that they *regain their autonomy as much as possible.*

So, interestingly, while coercive measures are usually considered to be detrimental to the autonomy of the patient, in the regime of the Wvggz coercive measures are (at least in some cases) considered to be meant to restore autonomy. The assumption seems to be that a severe mental illness may compromise a person’s autonomy, and consequently, by restoring the person’s mental health, autonomy may be restored (on autonomy and coercive measures, see Prinsen and Van Delden^8^). This may also be relevant for people who are not competent to decide about treatment of a physical illness due to their mental disorder. In such cases, the coercive measure can be aimed at restoring competency so the patient can make a competent decision about the treatment of the physical illness.^9^

The new legal regime has in practice resulted in an increase in bureaucracy and paperwork. For example, Van Melle et al. write that “obtaining legal authorization for providing compulsory care based on an SBD remains subject to highly complex and lengthy formal procedures”.^7^ Barkhof & Niele write: “the law is too complex, and the administrative burden is enormous. The information obligation includes too many letters to the patient, which frustrates both the patient and the care provider, is at the expense of contact and is very expensive.”^4^ They conclude: “While the Legislature intended to improve the legal position with the introduction of the Wvggz and Wzd, this was not achieved properly in the implementation.”^4^ It is our impression that their view about the new law is shared by many. Still, core ideas behind the law are laudable, and the inclusion of restoration of autonomy as a goal can be considered valuable.

## Conclusions

In 2020, a new legal regime on compulsory care in mental health came into effect in the Netherlands. One of its core aims was to strengthen the position of the patient. Notably, the law also explicitly recognizes that coercive measures may be taken not only for the purpose of preventing harm but also to restore a patient’s autonomy. This is interesting, as coercive measures may usually be conceived of as diminishing a patient’s autonomous choice, while here they are meant to increase a patients’ ability to make autonomous decisions about their future treatment and their lives in general. We feel that this is a valuable idea. Still, the law has become an administrative burden, taking a lot of time—which cannot be spent providing actual care and real contact and support to the patient. In a way this illustrates that it can be challenging to translate valuable ideas in effective laws, especially in a field as complex as mental healthcare.
